# Metabolic engineering of *Pichia pastoris* for sustainable production of 1,8-cineole from methanol

**DOI:** 10.3389/fmicb.2026.1866106

**Published:** 2026-06-19

**Authors:** Cui Zhao, Tengfei Wang, Hongling Liu, Yanpo Li, Weiwei Wang, XiHui Wang

**Affiliations:** 1 Food Nutrition and Health Research Laboratory, School of Health and Elderly Care, Shandong Women’s University, Jinan, China; 2 State Key Laboratory of Biobased Material and Green Papermaking (LBMP), Qilu University of Technology (Shandong Academy of Sciences), Jinan, China; 3 Key Laboratory of Shandong Microbial Engineering, College of Bioengineering, Qilu University of Technology (Shandong Academy of Sciences), Jinan, China

**Keywords:** 1, 8-Cineole, formaldehyde toxicity, metabolic engineering, methanol assimilation, *Pichia pastoris*

## Abstract

Methanol is an ideal feedstock for biomanufacturing and can be produced in large quantities from carbon dioxide via photocatalysis or electrolytic reduction, contributing to global carbon neutrality. In this study, we described an engineered *Pichia pastoris* strain capable of efficiently producing the monoterpene 1,8-cineole from methanol. First, a remarkable high-level synthesis of 1,8-cineole was achieved in *Pichia pastoris* by introducing an exogenous 1,8-cineole synthase, strengthening the MVA pathway, and suppressing the competing pathway. Subsequently, we significantly improved the yield of 1,8-cineole by reconstructing the methanol assimilation pathway and regulating the non-oxidative branch of the pentose phosphate pathway. To mitigate formaldehyde toxicity and redirect carbon flux toward central carbon metabolism for enhanced cellular vitality, the conversion of formic acid to serine was strengthened by overexpressing the *ADE3* and *SHM2* genes, resulting in an 82.2% increase in 1,8-cineole yield. Finally, we obtained 386.3 mg/L of 1,8-cineole from methanol in a 5 L bioreactor. This study established a metabolic engineering platform for the efficient synthesis of 1,8-cineole in *Pichia pastoris* based on methanol assimilation, providing a new strategy for the green biomanufacturing of terpenoids and offering a reference for the high-value utilization of methanol in synthetic biology.

## Introduction

1,8-cineole, a monoterpene compound widely present in the essential oils of *Eucalyptus*, camphor, rosemary, and other plants, imparts a refreshing aroma and exhibits diverse biological activities (Belcadi et al., 2023; Wang et al., 2026). In the field of medicine, 1,8-cineole can be used to treat respiratory infections and inflammatory diseases due to its antibacterial, anti-inflammatory, cough-suppressing, and bronchodilator effects (Ahmadi et al., 2026; Juergens et al., 2020). Another prospective application is its utilization as an insect repellent and grain protectant for grain storage (Chen et al., 2026; Krishanthi Abeywickrama et al., 2006). Despite exhibiting multiple promising activities, their low abundance in *Eucalyptus* species limits their application. At present, 1,8-cineole is extracted directly from *Eucalyptus* species through distillation or chemically synthesized from starting materials such as pinene, terpineol, or turpentine (Ben Salha et al., 2021; Kubota et al., 1998). With the rapid advancement of synthetic biology technologies, the *de novo* synthesis of natural products using microbial cell factories has emerged as an effective approach to overcome the limitations of traditional production methods. The most notable examples are the biosynthesis of amorphadiene and artemisinic acid, both of which are key precursors for the antimalarial drug artemisinin (Martin et al., 2003; Ro et al., 2006).

In nature, monoterpenes with a C10 skeleton are synthesized by terpene synthases from geranyl diphosphate (GPP). GPP is formed by farnesyl diphosphate (FPP) synthase through the coupling of three C5 units: two molecules of isopentenyl pyrophosphate (IPP) and one molecule of dimethylallyl pyrophosphate (DMAPP). IPP and DMAPP can be generated via the mevalonate (MVA) pathway or the 2-C-methyl-D-erythritol-4-phosphate (MEP) pathway. Currently, the biosynthesis of 1,8-cineole from sugars has been successfully achieved using *Yarrowia lipolytica* and Cyanobacteria as microbial chassis-based (Bhoir et al., 2025; Li et al., 2026; Sakamaki et al., 2023). However, despite playing a pivotal role in the cellular metabolism of most microorganisms used in industrial biotechnology, sugar cannot be economically derived from lignocellulose (Sonntag et al., 2015a). The growing global population requires sugars for food and nutrition. Consequently, the utilization of non-food carbon sources holds significant appeal for industrial biotechnology. Methanol is a promising one-carbon feedstock for the biomanufacturing of chemicals, and is regarded as an ideal “third-generation biorefinery” substrate for biomanufacturing due to its high energy density, ease of transportation, and low cost advantages (Jiang et al., 2025). Therefore, the microbial conversion of methanol into chemicals and other value-added products has emerged as a frontier in metabolic engineering and synthetic biology research. In recent years, increasing research has focused on converting methanol into high-value-added products using *Pichia pastoris* as a microbial chassis-based system (Guo et al., 2022; Nan et al., 2026; Qi et al., 2025; Zang et al., 2025b). Formaldehyde produced in the methanol assimilation process can combine with the intermediate products of sugar metabolism to enter the central carbon metabolism, generating NADPH at the same time, which provides additional carbon sources and reducing power for terpene synthesis and is expected to significantly increase the yield of terpenoids. Therefore, viable engineering strategies are needed to precisely regulate methanol metabolism in order to achieve the production of 1,8-cineole using methanol as the substrate.

**FIGURE 1:**
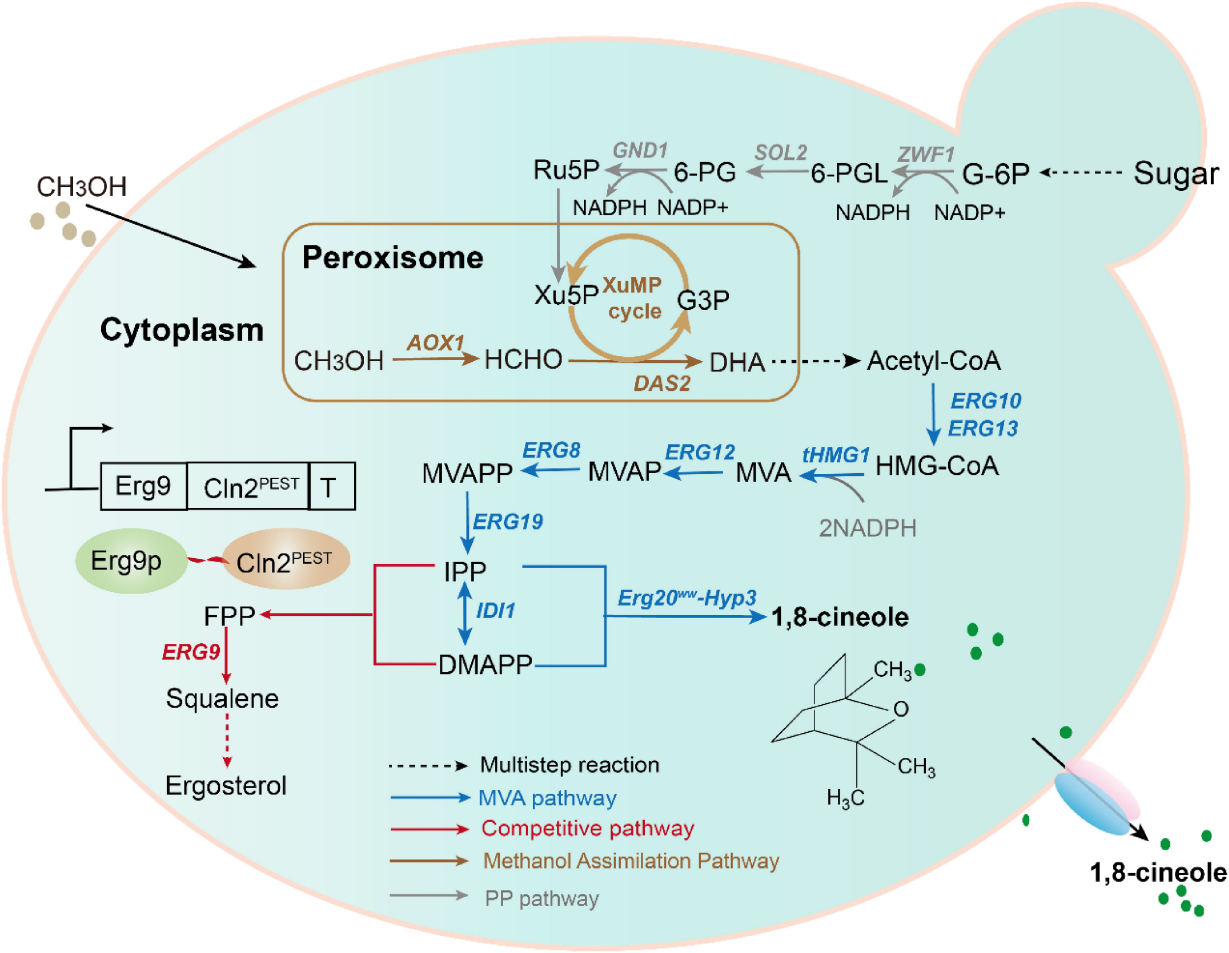
Schematic diagram of the production of 1,8-cineole from methanol. Acetyl-CoA, acetyl coenzyme A; HMG-CoA, 3-hydroxy-3-methyl-glutaryl coenzyme A; MVA, mevalonate; IPP, isopentenyl pyrophosphate; DMAPP, dimethylallyl pyrophosphate; GPP, geranyl diphosphate; FPP, farnesyl diphosphate; ERG10, acetyl-CoA C-acetyltransferase; ERG13, hydroxymethylglutaryl-CoA synthase; tHMG1, a truncated version of HMG-CoA reductase; ERG12, mevalonate kinase; ERG8, phosphomevalonate kinase; ERG19, diphosphomevalonate decarboxylase; IDI1, IPP isomerase; *Erg20*^ww^-*Hyp3*, *S. cerevisia*-derived mutant *Erg20*^F96W–N127W^ fused with a 1,8-cineole synthases gene (*Hyp3*).

In this study, we successfully achieved the *de novo* biosynthesis of 1,8-cineole by introducing a heterologous 1,8-cineole synthase and enhancing the MVA pathway while down-regulating the competing pathway. Furthermore, the methanol assimilation module and the non-oxidative branch of the pentose phosphate pathway were engineered to improve the efficiency of methanol assimilation. Then, by boosting the efficiency of formaldehyde assimilation and reducing the toxicity of formaldehyde to the cells and increasing cell viability, we ultimately achieved a 1,8-cineole yield of 386.3 mg/L in a 5-L bioreactor (Figure 1). This study investigated the application of one-carbon compounds in the synthesis of the natural product 1,8-cineole, representing the highest titer of 1,8-cineole produced in *Pichia pastoris* using sustainable carbon sources to date.

## Materials and methods

### Strains and culture conditions

In this study, *Escherichia coli* (*E. coli*) JM109 was used for plasmid construction and was cultivated at 37 °C in Luria Bertani (LB) liquid medium (10 g/L peptone, 5 g/L yeast extract, 10 g/L NaCl). The antibiotic ampicillin 100 μg/mL was supplemented to the medium when necessary. The *P. pastoris* GS115 (His4^–^, Mut^+^) was used for construction of 1,8-cineole-producing strains and cultivated in YPD (20 g/L peptone, 10 g/L yeast extract, and 20 g/L glucose) or YPM medium (20 g/L peptone, 10 g/L yeast extract, and 10 g/L methanol) at 30 °C with shaking at 250 rpm.

### DNA manipulation

All the strains, plasmids and primers used in this study are listed in Table 1 and Supplementary Tables 1, 2, respectively. The CRISPR-Cas9 method was employed for gene editing in *P. pastoris*. The donor DNA contained a promoter, the target gene, and a terminator. This donor DNA comprised a 1000-bp homologous arm upstream and downstream of the integration site. Then, a 20-bp guide sequence of target gene was analyzed by bioinformatics tool SgRNACas9^1^, and then the SgRNA gene was synthesized and ligated into a pMY-Sg plasmid by GENEWIZ Biotechnology Co., Ltd., (Suzhou, China) to generate single-guide RNA targeting plasmid. Finally, the donor DNA, SgRNA, and Cas9 cassette were then co-transformed into *P. pastoris* GS115 using electro transformation. All the genes involved in this study originate from *P. pastoris* unless otherwise specified.

**TABLE 1:** The main strains used in this study.

Strains	Relevant characteristics	Source
*E. coli* JM109	Wild-type	Lab stored
GS115	Mut^+^, *HIS4*^–^, *AOX1*	Lab stored
GS_0	GS115, *HIS4*::P*_*GAP1*_*-*RAD52*-T*_*AOX1*_ PNSI-2*::P*_*GAP1*_*-*Cas9*-T*_*AOX1*_*	Lab stored
GS_CnSA	GS_0, *PNSI-1*::P*_*AOX1*_*-*CnSA*-T*_*AOX1*_*	This study
GS_Hyp3	GS_0, *PNSI-1*::P*_*AOX1*_*-*Hyp3*-T*_*AOX1*_*	This study
GS_Hyp3_01	GS_Hyp3, *PNSII-1*::P*_*DAS2*_*-*Erg20*^ww^-T*_*AOX1*_*	This study
GS_Hyp3_02	GS_Hyp3, *PNSII-1*::P*_*DAS2*_*-*Erg20*^ww^-*Hyp3*-T*_*AOX1*_*	This study
GS_Hyp3_03	GS_Hyp3, *PNSII-1*::P*_*DAS2*_*-*Hyp3*-*Erg20*^ww^-T*_*AOX1*_*	This study
GS_Hyp3_04	GS_Hyp3_03, *PNSII-8*::P*_*TDH3*_*-*Erg10*-T*_*AOX1*_*-P*_*TEF1*_*-*Erg13*-T*_*AOX1*_*	This study
GS_Hyp3_05	GS_Hyp3_04, *PNSIV-7*::P*_*GAP1*_*-*tHMG1*-T*_*AOX1*_*	This study
GS_Hyp3_06	GS_Hyp3_05, *PNSIII-6*::P*_*ADH2*_*-*Erg12*-T*_*GAP1*_*-P*_*TEF1*_*-*Erg8*-T*_*DAS1*_*-P*_*GAP1*_*-*Erg19*-T*_*AOX1*_*	This study
GS_Hyp3_07	GS_Hyp3_06, *PNSII-5*::P*_*GPM1*_*-*IDI1*-T*_*AOX1*_*	This study
GS_Hyp3_08	GS_Hyp3_07, Erg9::P*_*HXT1*_*-Erg9-GGGGS-Cln2^PEST^-T*_*AOX1*_*	This study
GS_Hyp3_09	GS_Hyp3_08, *PNSIII-8*::P*_*ADH2*_*-*AOX1*-T*_*GAP1*_*-P*_*TEF1*_*-*DAS2*-T*_*TEF1*_*	This study
GS_Hyp3_10	GS_Hyp3_09, *PNSIV-12*::P*_*TEF1*_*-*ZWF1*-T*_*TEF1*_*-P*_*AOX1*_*-*SOL2*-T*_*DAS1*_*-P*_*DAS2*_*-*GND1*-T*_*AOX1*_*	This study
GS_Hyp3_11	GS_Hyp3_09, *PNSIV-12*::P*_*TEF1*_*-*RPE1*-T*_*TEF1*_*-P*_*AOX1*_*-*TAL1*-T*_*DAS1*_*-P*_*DAS2*_*-*TKL1*-T*_*AOX1*_*	This study
GS_Hyp3_12	GS_Hyp3_11, *FDH*::P*_*AOX1*_*-*ADE3*-T*_*TEF1*_*-P*_*AOX1*_*-*SHM2*-T*_*DAS1*_*	This study

### Fed-batch fermentation in shake flasks

For fed-batch fermentation in shake flasks, a single colony of the engineered strain was first selected and incubated in a 30 mL shake flask containing 10 mL YPD medium. The culture was grown at 30 °C and 250 rpm for 24 h to obtain the seed culture. Then, transfer the seed culture to a 250 mL shake flask containing 50 mL of YPD or YPM medium to achieve an initial OD_600_ of 0.2 or 0.5, respectively. The shake flask was cultivated under the conditions of 30 °C and 250 rpm for about 96 h. During the initial stage of the fermentation process, 10% (v/v) n-dodecane was added to the shake flasks to capture volatile 1,8-cineole. For the YPM medium culture, 10 g/L fresh methanol was added every 24 h.

### Fed-batch fermentation in bioreactors

For fed-batch fermentation in bioreactors, a single colony of the engineered strain was first selected and cultured in a 30 mL shake flask containing 10 mL YPD medium at 30 °C and 250 rpm for 24 h to obtain the primary seed culture. Then, 2 mL (1%) of the primary seed culture was transferred to a 500 mL shake flask containing 200 mL of YPD medium and cultured at 30 °C and 250 rpm for 24 h to obtain the secondary seed culture. The above secondary seed culture was inoculated into a 5 L bioreactor at a 10% inoculum volume for fed-batch fermentation for approximately 144 h, with a working volume of 3 L. During the fermentation process, the dissolved oxygen level was maintained at 30%, the agitation speed was adjusted in accordance with cell growth (600–800 rpm), aeration rate was 2.0 vvm, and the pH was maintained at 6.0 by adding NH_3_⋅H_2_O. The fermentation process consists of two phases. In the first phase, 20 g/L glycerol was fed into the bioreactor to sustain cell growth. After glycerol depletion, the process transitioned to the methanol feeding phase. In the 5 L bioreactor, 10 g/L of fresh methanol was added every 24 h.

### Quantification of 1,8-cineole, squalene

For the quantitative detection of 1,8-cineole, 10 mL of the fermentation broth was centrifuged at 5000 *g* for 10 min to obtain the organic phase, supernatant, and cell pellet. The organic phase was dried with anhydrous MgSO_4_ and then filtered through a 0.45 μm syringe filter for the quantitative determination of 1,8-cineole. Furthermore, add 0.5 mL of quartz sand to the cell pellet and the mixture was dispersed using a homogenizer. Then, an equal volume of *n*-hexane (≥99%) (0.5 mL) was added as an extraction solvent, and the mixture was centrifuged at 12 000 *g* for 3 min for the quantitative detection of intracellular squalene. Quantification and identification were performed by using a mass spectrometer (GC-MS-Trace1310-ISQ LT, Thermo Fisher Scientific) equipped with an HP-5 ms capillary column (30 m × 0.25 mm × 0.25 μm). The sample analysis procedure is as follows: the oven temperature was initially held at 80 °C for 3 min, sequentially increased at a rate of 20 °C/min to 270 °C, and held for 6 min. The detector temperature was maintained at 300 °C. The injection volume was 1 μL, the split ratio was 20:1, and the flow rate was 1 mL/min. 1,4-cineole (CAS: 470-67-7) was adopted as the internal standard for the quantitative determination of 1,8-cineole. The standard curve for 1,8-cineole and squalene were established using purchased 1,8-cineole standard (CAS:470-82-6, Sigma-Aldrich, China; Supplementary Figure 1) and squalene standard (CAS: 111-02-4, Sigma-Aldrich, China; Supplementary Figure 2).

### Quantitative detection of ergosterol

To determine intracellular ergosterol content, take 10 mL of culture medium and centrifuge at 6000 *g* for 5 min, then discard the supernatant. Wash the cell pellet with sterile water, then treat with 2 mL of 50% KOH (v/v) and 1.5 mL of anhydrous ethanol at 88 °C for 3 h. After cooling to room temperature, add petroleum ether and vortex for 10 min. After standing until layers separate, aspirate the oil-ether layer and dilute appropriately. Measure the absorbance at 281.5 nm using a spectrophotometer. Ergosterol content was quantified in milligrams of ergosterol per gram of dry cell weight (mg/g Dry Cell Weight). The ergosterol standard curve was shown in Supplementary Figure 3 (CAS: 57-87-4, Sigma-Aldrich, China).

### Measurement of biomass, methanol, formaldehyde

OD_600_ was measured by an Orion AquaMate 8000 UV–visible spectrophotometer (Thermo Scientific, Shanghai, China). Dry cell weight (DCW) measurements were performed by centrifuging (8000 *g*, 5 min) a 10 mL fermentation broth in preweighed dry test tubes to harvest cells. The cell pellet was then washed and dried at 60 °C until constant weight and then weighed. DCW = OD_600_ × 0.40.

Methanol was analyzed by HPLC with a BioRad Aminex HPX-87H column (300 mm × 7.8 mm, Bio-Rad, Hercules, CA) at 60 °C with 5 mM H_2_SO_4_ at a flow rate of 0.6 mL/min and a refractive index (RI) detector. Formaldehyde concentration was measured by the Nash reaction method: cells were centrifuged at 12 000 *g* for 10 min and the cells were disrupted by transferring 125 μL of the supernatant to a well. 125 μL of Nash reagent (5 M NH_4_OAc and 50 mM acetylacetone) were added to each well and incubated at 37 °C for 1 h, and the absorbance was measured at 412 nm using a spectrophotometer (Thermo Scientific, Shanghai, China). The measured formaldehyde concentration was then normalized to the OD_600_ of the corresponding timepoint.

## Results

### Biosynthesis of 1,8-cineole in *P. pastoris*

**FIGURE 2:**
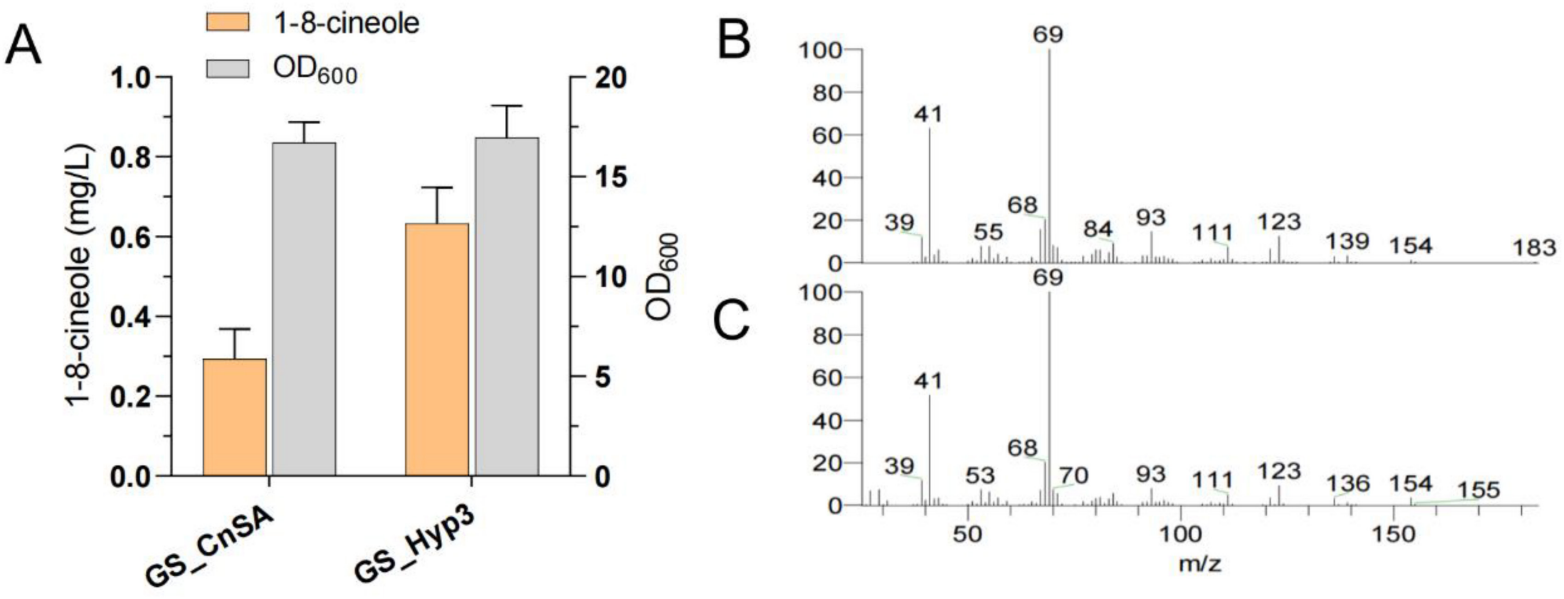
Preliminary synthesis of 1,8-cineole in *P. pastoris*. **(A)** 1,8-Cineole titer and biomass of engineered strains GS_CnSA and GS_Hyp3. **(B)** Mass spectrometry analysis of 1,8-cineole produced by the engineered strain GS_Hyp3. **(C)** Mass spectrometric analysis of 1,8-Cineole standard (CAS:470-82-6, Sigma-Aldrich, China). These data represent average values and standard deviations achieved from three independent experiments.

To achieve the biosynthesis of 1,8-cineole in *Pichia pastoris* (*P. pastoris*), we selected the 1,8-cineole synthases from *Streptomyces clavuligerus* (Bhoir et al., 2025) and *Hypoxylon* sp. (Li et al., 2026; Shaw et al., 2015), which are encoded by the genes *CnSA* and *Hyp3*, respectively (the original nucleotide sequences are shown in Supplementary Table 3). Subsequently, the codon-optimized genes *CnSA* and *Hyp3* were integrated into the *P. pastoris* GS115 genome to generate the engineered strains GS_CnSA and GS_Hyp3, respectively. The engineered strains were cultivated in YPM medium containing 1% methanol and covered with 10% (v/v) n-dodecane to capture volatile 1,8-cineole (Jiang et al., 2017). The results showed (Figure 2A) that the 1,8-cineole production of GS_CnSA and GS_Hyp3 was 0.29 mg/L and 0.63 mg/L, respectively, and no significant growth impairment was observed. The product was identified by GC-MS as 1,8- cineole (Figures 2B,C), with a mass-to-charge ratio (m/z) of 154 (Figures 2B,C) and a retention time of 7.66 min. Therefore, strain GS_Hyp3 was selected as the starting strain for the subsequent engineering design.

### Optimization of the MVA pathway for enhanced synthesis of 1,8-cineole

**FIGURE 3:**
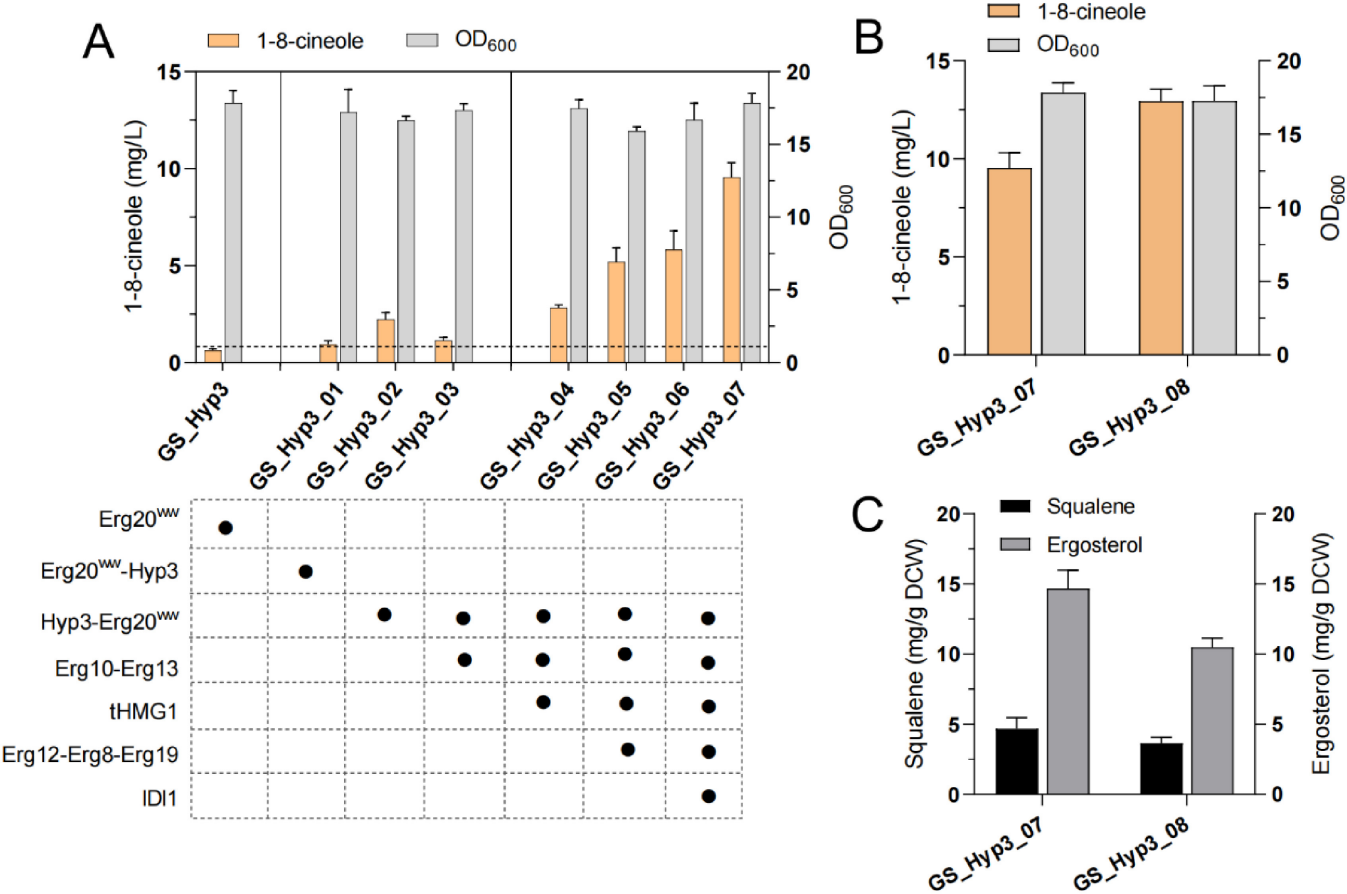
Optimizing the MVA pathway and mitigating the competitive pathway to enhance 1,8-cineole synthesis. **(A)** The 1,8-cineole titer and biomass of engineered strains GS_Hyp3_01 to GS_Hyp3_08, with strain GS_Hyp3 as the control (dotted line). **(B)** 1,8-cineole titer and biomass of engineered strains GS_Hyp3_07 and GS_Hyp3_08. **(C)** The intracellular squalene and ergosterol contents of the engineered strains GS_Hyp3_07 and GS_Hyp3_08. These data represent average values and standard deviations achieved from three independent experiments.

The low GPP pool in yeast is a critical factor limiting monoterpene synthesis and resulting in low monoterpene yields. Therefore, to overcome this limitation, we introduced the *Erg20* mutant *Erg20^F96W–N127W^* (*Erg20^ww^*) (Blanchard and Karst, 1993; Ignea et al., 2014) to increase the availability of the GPP pool used for synthesizing 1,8-cineole. Then we introduced the mutant *Erg20^ww^* into strain GS_Hyp3 to alleviate the competition between FPP and GPP, thereby generating strain GS_Hyp3_01. The results showed that strain GS_Hyp3_01 achieved a 1,8-cineole yield of 0.9 mg/L (Figure 3A), representing an 47.6% increase compared to the initial strain GS_Hyp3. Additionally, protein fusion strategies are widely employed to enhance enzyme catalytic activity and reduce byproduct formation (Wang et al., 2011). Consequently, the fusion genes *Erg20^ww^*-*Hyp3* and *Hyp3-Erg20^ww^* were integrated into the *P. pastoris* GS115 genome, yielding strains GS_Hyp3_02 and GS_Hyp3_03 (linker peptide of GGGS), respectively. As shown in Figure 3A, the 1,8-cineole yield of GS_Hyp3_02 reached 2.2 mg/L, exhibiting a 1.4-fold improvement over the 1,8-cineole yield of the control strain GS_Hyp3_01. Furthermore, overexpression of genes associated with the MVA pathway promoted 1,8-cineole production to varying degrees, particularly when overexpressing the rate-limiting genes *IDI1* (IPP isomerase gene) and *tHMG1* (a truncated version of HMG-CoA reductase). Based on the above, the 1,8-cineole yield of the MVA pathway-enhanced strain GS_Hyp3_07 reached 9.6 mg/L (Figure 3B).

Traditional terpenoid precursors are tightly coupled with ergosterol biosynthesis. However, ergosterol is essential for yeast survival and cannot be reduced by knocking out the squalene synthase gene (Erg9) to decrease competition from the sterol pathway (Cao et al., 2018; Vickers et al., 2017). Erg9 dysfunction leads to ergosterol depletion and cell growth penalties. Protein degradation strategies represent a potentially powerful tool in metabolic engineering for studying functional defects at metabolic nodes (Cheah et al., 2023; Lu et al., 2021; Peng et al., 2017, 2018). Therefore, we engineered protein degradation to reduce the consumption of monoterpene synthesis flux by squalene production. The degradation peptide Cln2*^PEST^* from *S. cerevisiae* was cloned and fused to Erg9 at its C-terminus via the linker GGGGS (Peng et al., 2017). Then, the expression cassette was integrated into the GS_Hyp3_07 genome to form strain GS_Hyp3_08. The results showed that the intracellular squalene and ergosterol contents of the engineered strain GS_Hyp3_08 decreased by 22.4% and 28.5%, respectively (Figure 3C). This indicated that destabilization of Erg9p was successfully achieved, accompanied by a 35.5% increase in the target product 1,8-cineole, reaching 12.9 mg/L (Figure 3B). Additionally, no growth impairment was observed in strain GS_Hyp3_08 compared to the control strain GS_Hyp3_07.

### Fine-tuning the PP pathway to enhance methanol assimilation

Methanol is a promising substrate for biomanufacturing due to its low cost and non-food competition (Schrader et al., 2009). The utilization of non-food carbon sources holds significant appeal for industrial biotechnology. However, the bioconversion of methanol faces major challenges, including the toxicity of the intermediate metabolite formaldehyde and carbon losses during metabolic processes (Sonntag et al., 2015a).

**FIGURE 4:**
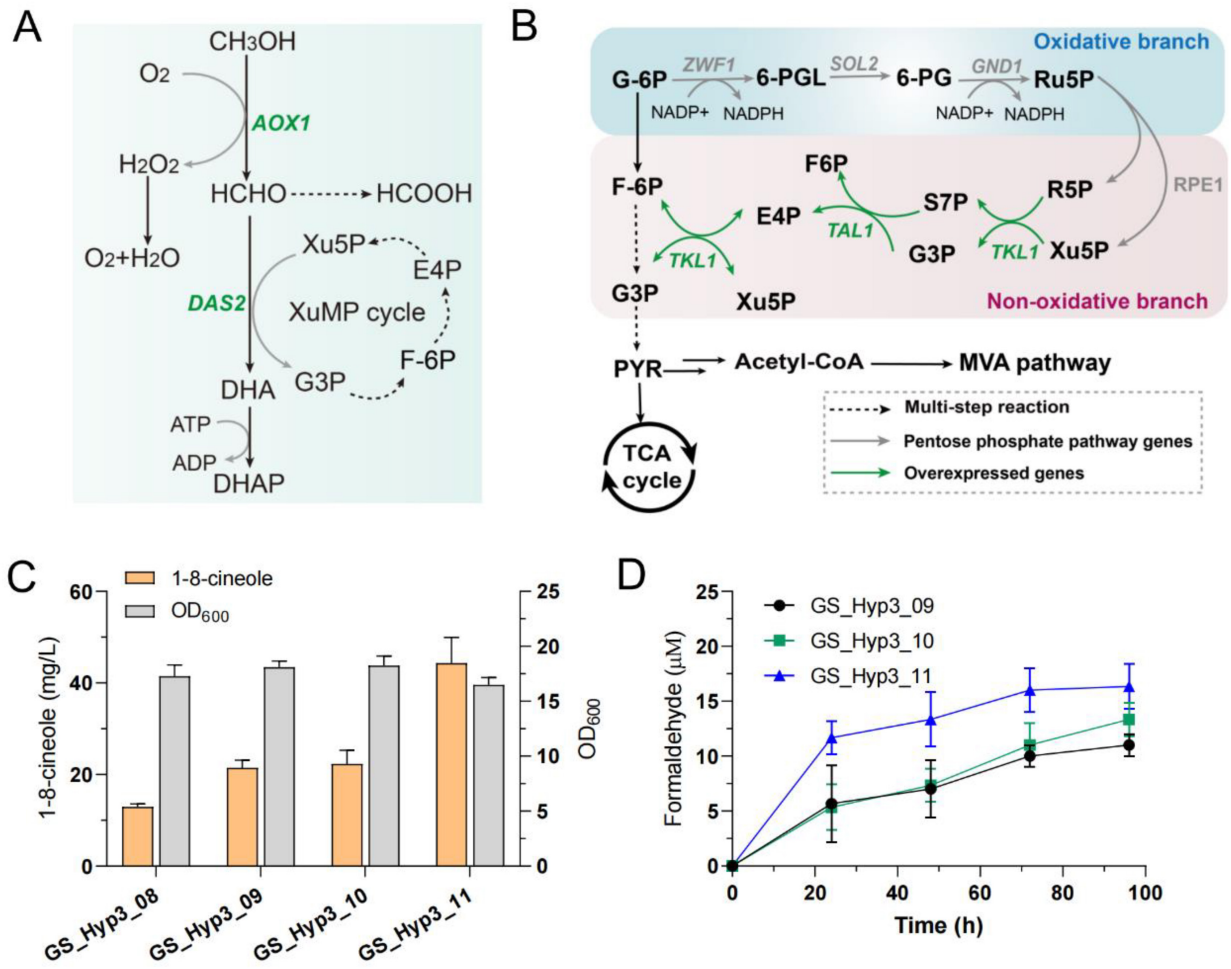
Enhancing methanol assimilation to boost 1,8-cineole production. Schematic diagram of the regulation of the methanol assimilation pathway **(A)** and the PP pathway **(B)** *AOX1*, alcohol oxidase; *DAS2*, Dihydroxyacetone synthase; *ZWF1*, glucose-6-phosphate dehydrogenase; *SOL2*, 6-phosphogluconolactonase; *GND1*, phosphogluconate dehydrogenase; *RPE1*, ribulose-phosphate 3-epimerase; *TKL1*, transketolase; *TAL1*, transaldolase; G-6P, glucose-6-phosphate; F-6P, fructose-6-phosphate; G3P, glyceraldehyde-3- phosphate; PYR, pyruvate; Ru5P, ribulose 5-phosphate; R5P, ribose-5-phosphate; Xu5P, xylose-5-phosphate; S7P, sedoheptulose-7-phosphate; and E4P, erythrose-4-phosphate. **(C)** The 1,8-cineole titer and biomass of engineered strains GS_Hyp3_08 to GS_Hyp3_11. **(D)** The intracellular formaldehyde content of engineered strains GS_Hyp3_09 to GS_Hyp3_11. These data represent average values and standard deviations achieved from three independent experiments.

In the natural methanol assimilation pathway of *P. pastoris*, methanol is converted to formaldehyde and H_2_O_2_ by alcohol oxidase (encoded by AOX1), and the latter is immediately decomposed by catalase (CAT) to protect the cells from potential toxicity or oxidative damage caused by H_2_O_2_ (Figure 4A). To enhance methanol metabolism, this study constructed an engineered strain GS_Hyp3_09 by integrating the AOX1 and dihydroxyacetone synthase gene (DAS2) into strain GS_Hyp3_08 through CRISPR/Cas9-mediated homologous recombination. To evaluate the production capacity of 1,8-cineole, the engineered strain GS_Hyp3_09 was grown in YPM medium containing 1% methanol. After 96 h of cultivation in medium with methanol as the sole carbon source, the engineered strain GS_Hyp3_09 produced 21.5 mg/L of 1,8-cineole, which is a 65.8% improvement compared to the control strain GS_Hyp3_08 (Figure 4C).

The pentose phosphate (PP) pathway is divided into an oxidative branch that produces pentose phosphates and a non-oxidative branch that consumes pentose phosphates. The oxidative branch is mainly responsible for generating NADPH (Rusz et al., 2021). Additionally, studies have shown that the genes associated with the PP pathway are significantly upregulated in the context of methanol metabolism (Gao et al., 2022; Rußmayer et al., 2015). In the oxidation branch of the PP pathway, G-6P is converted to Ru5P via the enzymes *ZWF1*, *SOL2*, and *GND1*. Subsequently, Ru5P is converted to Xu5P by RPE1, which is the key intermediate involved in methanol metabolism (Figure 4B). This suggests that enhancing the PP pathway not only provides additional NADPH and precursor Xu5P, but also has the potential to improve methanol metabolic efficiency and increase 1,8-cineole production. Based on the above, strain GS_Hyp3_10 was obtained by overexpressing the genes *ZWF1*, *SOL2*, and *GND1* in strain GS_Hyp3_09. Unfortunately, the 1,8-cineole yield of the engineered strain GS_Hyp3_10 showed only a limited improvement of 4.1%, reaching 22.3 mg/L.

The non-oxidative branch of PP pathway is responsible for converting pentoses such as R5P and Xu5P into intermediates (F-6P and GAP) of glycolysis, thereby linking carbon metabolism networks. The key enzymes at this stage include ribose-5-phosphate epimerase (RPE1), transaldolase (TAL1) and transketolase (TKL1), which are encoded by genes *RPE1*, *TAL1* and *TKL1*, respectively (Figure 4B). Therefore, we constructed the *RPE1-TAL1-TKL1* expression cassette and integrated it into the genome of the engineered strain GS_Hyp3_09 to produce the engineered strain GS_Hyp3_11. The results showed that the 1,8-cineole yield of GS_Hyp3_11 reached 44.3 mg/L, which was significantly increased by 1.1-fold compared to the control strain GS_Hyp3_09 (Figure 4C). However, we observed that the formaldehyde concentration of strain GS_Hyp3_11 increased significantly by 32.2% compared to the control strain GS_Hyp3_09 (Figure 4D), suggesting that complex metabolic engineering may have led to metabolic congestion. Thus, enhancing formaldehyde assimilation could be a key strategy for potentially boosting 1,8-cineole production. The above results demonstrated that the non-oxidative branch of the PP pathway represents a key target for enhancing methanol assimilation and increasing target product yields.

### Enhancing formaldehyde assimilation to boost cell vitality

**FIGURE 5:**
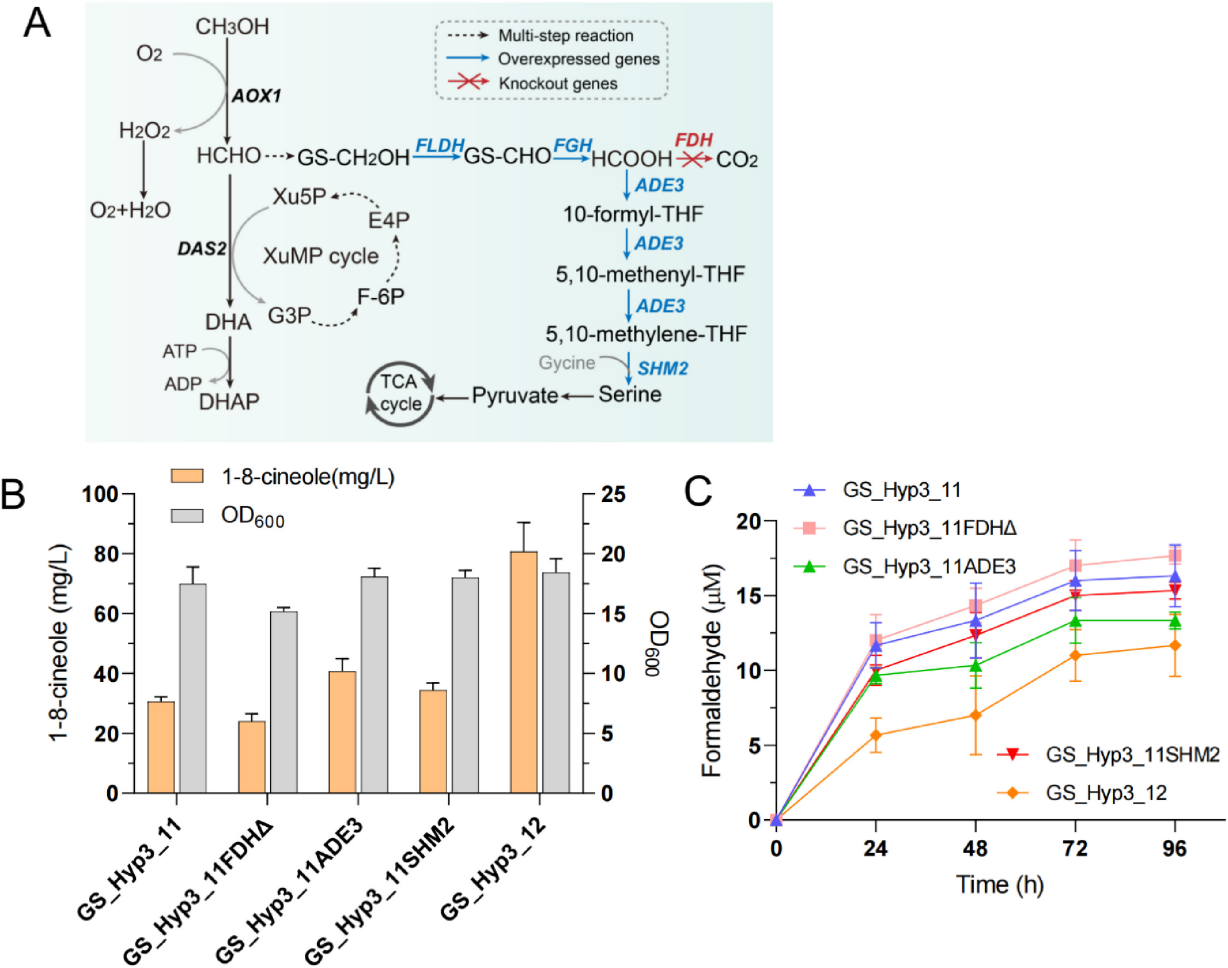
Enhancing formaldehyde assimilation to boost the biosynthesis of 1,8-cineole. **(A)** Schematic diagram of the enhanced formaldehyde assimilation pathway. *FLDH*, glutathione-dependent formaldehyde dehydrogenase; *FGH*, S-formylglutathione hydrolase; *FDH*, formate dehydrogenase; *ADE3*, trifunctional formate-tetrahydrofolate ligase; *SHM2*, serine hydroxymethyl transferase. **(B)** The 1,8-cineole titer and biomass of engineered strains GS_Hyp3_11, GS_Hyp3_11FDHΔ, GS_Hyp3_11ADE3, GS_Hyp3_11SHM2 and GS_Hyp3_12. **(C)** The intracellular formaldehyde content of engineered strains GS_Hyp3_11, GS_Hyp3_11FDHΔ, GS_Hyp3_11ADE3, GS_Hyp3_11SHM2 and GS_Hyp3_12. These data represent average values and standard deviations achieved from three independent experiments.

During the methanol assimilation process, methanol is oxidized to formaldehyde, which is then oxidized to CO_2_, resulting in carbon loss (Figure 5A). This process is catalyzed by a cascade of reactions involving glutathione-dependent formaldehyde dehydrogenase (FLDH), S-formylglutathione hydrolase (FGH), and formate dehydrogenase (FDH) (Figure 5A). However, evidence indicated that *FLDH*-deficient strains exhibit growth retardation (Zang et al., 2025a). Therefore, carbon loss cannot be remedied by simply knocking out the *FLDH* gene. During formaldehyde metabolism, formic acid is converted to CH2-THF through a three-step reaction catalyzed by trifunctional formate-tetrahydrofolate ligase (*ADE3*). Subsequently, CH2-THF is converted to serine via serine hydroxymethyl transferase (*SHM2*) with the participation of glycine, and then enters central carbon metabolism. To determine the individual contributions of each modification, we generated strains GS_Hyp3_11FDHΔ, GS_Hyp3_11ADE3, and GS_Hyp3_11SHM2 by knocking out the *FDH* gene, overexpressing the *ADE3* gene, and overexpressing the *SHM2* gene, respectively, in strain GS_Hyp3_11. The results showed that the growth of GS_Hyp3_11FDHΔ was significantly impaired, with biomass reduced by 13.4% compared to the control strain (Figure 5B). We speculate this may relate to an imbalance in NAD^+^/NADH levels. Since the FDH reaction produces NADH, the deletion of FDH leads to reduced NADH synthesis, which in turn downregulates intracellular oxidative phosphorylation, resulting in insufficient cellular energy and impaired growth. As expected, the yield of 1,8-cineole was 24.05 mg/L, a 21.6% decrease compared to the control strain. The formaldehyde levels in the GS_Hyp3_11FDHΔ strain were slightly higher than those in the control strain GS_Hyp3_11 (Figure 5C). We consider this to be one of the factors contributing to the impaired growth of the GS_Hyp3_11FDHΔ strain. Based on strain GS_Hyp3_11, ADE3 was overexpressed to generate strain GS_Hyp3_11ADE3. The results showed that formaldehyde levels in GS_Hyp3_11ADE3 decreased by 18.4% compared to strain GS_Hyp3_11, while growth was slightly improved, and 1,8-cineole increased by 32.7% to 40.7 mg/L (Figure 5B). Formaldehyde levels in GS_Hyp3_11SHM2 did not show a significant decrease compared to the control strain GS_Hyp3_11 (Figure 5C), and no growth impairment was observed. Regarding 1,8-cineole production, strain GS_Hyp3_11SHM2 produced 34.3 mg/L of 1,8-cineole, representing a modest 11.9% increase compared to the control strain (Figure 5B).

To further enhance formaldehyde assimilation and reduce carbon loss, the *FDH* gene was knocked out and the *ADE3* and *SHM2* genes were overexpressed in the GS_Hyp3_11 strain to obtain the GS_Hyp3_12 strain. The results showed that compared to the control strain GS_Hyp3_11, the engineered strain GS_Hyp3_12 exhibited a significant 23.1% reduction in formaldehyde concentration, and a significant 11.5% increase in biomass (Figures 5B,C). The reduction in formaldehyde concentration and increased biomass of the engineered strain GS_Hyp3_12 demonstrated that *ADE3* and *SHM2* accelerate the metabolism of formaldehyde, a toxic intermediate, into downstream pathways. Consequently, the titer of 1,8-cineole improved by 82.2%, reaching 80.7 mg/L. These results illustrate the potential for enhancing methanol conversion and increasing target product yields by boosting formaldehyde assimilation and reducing carbon loss.

### Production of 1,8-cineole in a 5 L bioreactor

**FIGURE 6:**
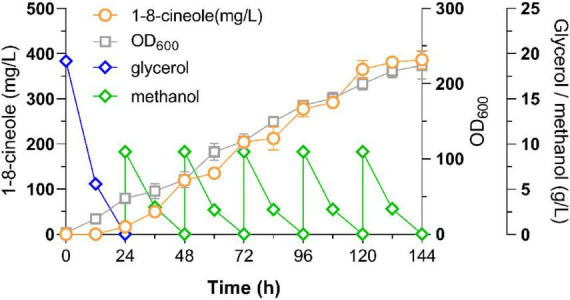
Fermentation of the engineered strain GS_Hyp3_12 in a 5 L bioreactor. These data represent average values and standard deviations achieved from three independent experiments.

To evaluate the fermentation performance and 1,8-cineole production capacity of the engineered strain GS_Hyp3_12, fed-batch fermentation was performed in a 5 L bioreactor using methanol. The fermentation process consists of two phases. In the first phase, 20 g/L glycerol was fed into the bioreactor to sustain cell growth. After glycerol depletion, the process transitioned to the methanol feeding phase. First, the engineered strain was grown in 20 g/L glycerol to accumulate biomass. As shown in Figure 6, the biomass of the engineered strain GS_Hyp3_12 rapidly accumulated during the initial 24 h. Subsequently, when glycerol was depleted, methanol was added to the bioreactor at a final concentration of 10 g/L to initiate the methanol feeding phase. For the synthesis of the target product, the titer of 1,8-cineole increased quickly after 24 h of the fermentation process and stabilized at 120–144 h. Finally, the engineered strain GS_Hyp3_12 achieved a 1,8-cineole yield of 386.3 mg/L from methanol, with an intracellular yield of 23.7 μg/L (Supplementary Figure 5). This study achieved *de novo* biosynthesis of the monoterpene 1,8-cineole in *P. pastoris* using pure methanol as a carbon source, demonstrating the significant potential of *P. pastoris* for the biosynthesis of monoterpenes from methanol.

## Discussion

In this study, we developed a sustainable method for synthesizing 386.3 mg/L of 1,8-cineole in a 5-L bioreactor from methanol using metabolically engineered *Pichia pastoris* as a microbial chassis-based.

The methylotrophic yeast *P. pastoris* possesses robust heterologous expression capabilities and a native MVA pathway. In the MVA pathway, the bifunctional enzyme (encoded by *Erg20*) is a key node in monoterpene synthesis, combining both geranyl diphosphate synthase (GPPS) and farnesyl pyrophosphate synthase (FPPS) activities and mediating the production of GPP from IPP and DMAPP. However, most of the GPP is then converted to FPP for the production of ergosterol and other primary and metabolite products. Therefore, the level of GPP used for monoterpene synthesis is extremely low. Ignea et al. (2014) converted the geranyl diphosphate synthase into a dominant negative form, so as to decrease the ability of the endogenous Erg20p to function as a FPPS, without entirely abolishing sterol biosynthesis. This design resulted in a significant increase in the yield of sabinene. In this work, the introduction of *Erg20*^ww^ resulted in a significant 47.6% increase in the 1,8-cineole titer. Subsequently, the introduction of the fusion gene *Erg20*^ww^-*Hyp3* (strain GS_Hyp3_02) resulted in a 1.4-fold increase in 1,8-cineole titer compared to the control strain GS_Hyp3_01. This suggests that *Erg20*^ww^ facilitates the redistribution of carbon flux at the critical juncture between the GPP and FPP. Furthermore, squalene synthase (encoded by *Erg9*) mediates the synthesis of squalene from FPP. Although we reduced the carbon flux of FPP via *Erg20*^ww^, *Erg9* remains a significant node in the synthesis of squalene and ergosterol, and competes with the synthesis of 1,8-cineole. Since ergosterol is essential for cell growth, inactivating *Erg9* could potentially lead to cell death. The degradation peptide Cln2^PEST^ induces the degradation of Erg9p (Peng et al., 2017), thereby reducing the competition between squalene and ergosterol for the 1,8-cineole synthesis pathway, resulting in a significant 35.5% increase in 1,8-cineole yield. Furthermore, we observed that the growth of the engineered strain GS_Hyp3_08 was not affected by the decrease in the content of squalene/ergosterol (Figure 3B). Furthermore, a comparison of methanol consumption between GS_Hyp3_07 and GS_Hyp3_08 revealed that the destabilization of Erg9 did not affect methanol metabolism (Supplementary Figure 4). The above results indicated that the Erg9p levels may naturally exceed those required for normal cell, allowing the cells to tolerate the decrease in Erg9p to a certain extent.

To enable the sustainable production of 1,8-cineole from methanol, the PP pathway was engineered to enhance the methanol assimilation capacity in *P. pastoris*. In the MVA pathway, the biosynthesis of terpenoids consumes a significant amount of NADPH. Overexpressing genes associated with the PP pathway can increase the supply of NADPH in yeast (Gao et al., 2022). However, the enhancement of the oxidative branch of the PP pathway (engineered strain GS_Hyp3_10) resulted in only a modest 4.1% increase in the 1,8-cineole titer. Previous reports have shown that methanol metabolism in *P. pastoris* can indirectly provide sufficient NADPH (Gao et al., 2022). Although the synthesis of 1,8-cineole consumes a large amount of NADPH, the remaining NADPH can maintain other metabolic pathways. Fortunately, the enhancement of the non-oxidative branch of the PP pathway resulted in a 1.1-fold increase in the 1,8-cineole titer of the engineered strain GS_Hyp3_11. In the non-oxidative branch of the PP pathway, RPE1 catalyzes the interconversion of Ru5P and Xu5P to maintain the balance of the pentose pool. TKL1 is the core enzyme of the non-oxidative branch of the PP pathway, catalyzing the reaction of Xu5P with R5P or E4P to produce GAP and S7P or F-6P. These intermediates subsequently enter central carbon metabolism to provide the carbon skeleton for methanol assimilation. The biosynthesis of monoterpenes uses GPP as a direct backbone and is highly dependent on the supply of acetyl-CoA, ATP, NADPH, and the phosphocarbon skeleton (Ye et al., 2024). The native XuMP pathway in *P. pastoris* consumes 3 molecules of ATP for every 1 molecule of G3P produced. This high ATP consumption leaves insufficient ATP available for the MVA phosphorylation step, potentially leading to downregulation of the MVA rate-limiting enzyme tHMGR due to ATP deficiency. Therefore, the next step will be to fine-tune the ATP supply to increase the yield of 1,8-cineole. Unlike XuMP, the heterologous RuMP pathway consumes only 1 ATP per molecule of G3P produced, significantly conserving ATP for the MVA phosphorylation step (Kuzman et al., 2026; Zang et al., 2025a). Therefore, the future engineering focus of this study will be on either completely replacing native XuMP with heterologous RuMP, or the construction of dual-pathway microbial cell factories, in which the native XuMP sustains basal cell growth and the high-efficiency methanol-assimilating RuMP acts as the core for methanol assimilation.

Formaldehyde is the primary oxidation product of methanol, and its accumulation within cells can produce toxicity. Previous studies have demonstrated that formaldehyde exhibits stronger toxicity than methanol (Sonntag et al., 2015b; Zhan et al., 2021). The formaldehyde concentration detection result of the engineering strain GS_Hyp3_11 indicated that reducing the accumulation of formaldehyde may be the key to further improving the yield of 1,8-cineole. The rapid assimilation of formaldehyde not only reduces the accumulation of toxic intermediates, but also provides a driving force for central carbon metabolism, restoring cellular vitality. Accordingly, we knocked out the FDH gene in the engineered strain GS_Hyp3_11 to block the conversion of carbon flux toward CO_2_. Next, the *ADE3* and *SHM2* genes were overexpressed (yielding strain GS_Hyp3_12) to redirect the carbon flux toward serine and subsequently into the central carbon metabolism, resulting in a significant 82.2% improvement in the 1,8-cineole titer, which reached 80.7 mg/L. In *P. pastoris*, *SHM2* and *ADE3* are key functional genes in the one-carbon metabolism compensatory pathway (indirect detoxification) (Anderson et al., 2002; Yasokawa et al., 2010). Although this pathway does not directly catalyze the degradation of formaldehyde, it mitigates formaldehyde toxicity through multiple mechanisms, including the remodeling of intracellular metabolic pools and homeostatic regulation, and thus constitutes a metabolic compensation mechanism under stress conditions. First, *ADE3* can expand the intracellular CH_2_-THF pool. High intracellular concentrations of CH_2_-THF can undergo non-enzymatic covalent binding with formaldehyde to form hydroxymethyl-THF derivatives, thereby reducing intracellular formaldehyde concentrations (Wit et al., 2023). Second, the glycine reduction pathway is activated to facilitate the diversion of formaldehyde carbon assimilation. After formaldehyde is non-enzymatically converted to CH_2_-THF, it is sequentially converted to glycine and serine under the catalysis of *SHM2*. These compounds ultimately enter the glycolytic pathway to produce pyruvate, thereby converting toxic formaldehyde carbon into intracellular biomass (Wang et al., 2025; Xing et al., 2018). In summary, *ADE3* and *SHM2* lay the groundwork for indirectly reducing the toxicity of formaldehyde and enabling *P. pastoris* to robustly utilize methanol as a carbon source for producing high-value-added chemicals.

Currently, the highest yield of 1,8-cineole has been reported at 755.39 mg/L in *Y. lipolytica* (Li et al., 2026). Both *P. pastoris* and *Y. lipolytica* are non-conventional GRAS grade yeasts and possess a complete endogenous MVA pathway. However, the two strains exhibit significant differences in chassis adaptability for 1,8-cineole synthesis. The methanol carbon source pathway unique to *P. pastoris* relies on the peroxisomal XuMP pathway to utilize methanol as the sole carbon source. Furthermore, since methanol metabolism in *P. pastoris* is concentrated in the peroxisomes, the MVA pathway can be targeted to the peroxisomes to *in situ* enrich methanol-derived acetyl-CoA and NADPH, thereby achieving a stable increase in the yield of monoterpene (Gao et al., 2024). In addition, methanol is a low-cost non-food C1 carbon source with a simple molecular structure and a concise metabolic pathway. Meanwhile, methanol can be produced in a fully carbon-neutral process by coupling photocatalysis with electrocatalytic CO_2_ conversion. The high acetyl-CoA flux and PP pathway flux in *Y. lipolytica* naturally suit the monoterpene synthesis via the MVA pathway (Czajka et al., 2018). Fatty acid metabolism can regenerate NADPH via the PP pathway, perfectly meeting the cofactor requirements for 1,8-cineole synthesis. Second, the broad carbon substrate range of *Y. lipolytica* enables it to utilize waste cooking oil, fatty acids, and other substances as exclusive carbon sources, facilitating the resource recovery of waste. In summary, we conclude that *P. pastoris* is better suited for low-carbon methanol-based synthesis and holds significant industrial potential following the introduction of the RuMP pathway and mitigation of CO_2_ consumption. *Y. lipolytica* is better suited for the valorization of waste lipids and represents an excellent chassis for low-cost biosynthesis of monoterpenes. Finally, the engineered strain GS_Hyp3_12 achieved a 1,8-cineole yield of 386.3 mg/L from methanol in a 5-L bioreactor. To the best of our knowledge, this represents the highest titer of 1,8-cineole produced in *P. pastoris* using sustainable carbon sources to date.

## Conclusion

This study demonstrated a sustainable method for synthesizing 1,8-cineole from methanol using metabolically engineered *Pichia pastoris* as a microbial chassis-based. The *de novo* synthesis of 1,8-cineole was initially achieved by introducing an exogenous 1,8-cineole synthase. Furthermore, the yield of 1,8-cineole was significantly increased by enhancing the MVA pathway and suppressing the competitive pathway. Methanol assimilation was then enhanced by engineering the methanol assimilation pathway and the non-oxidative branch of the PP pathway. Thereafter, by strengthening formaldehyde assimilation to mitigate its cytotoxicity, the 1,8-cineole titer reached 386.3 mg/L in a 5-L bioreactor. This study explored the application of one-carbon compounds in the synthesis of natural products, providing new ideas for the precise regulation of the methanol metabolic flow and offering a reference for the green synthesis of other terpenoids.

## Data Availability

The datasets presented in this study can be found in online repositories. The names of the repository/repositories and accession number(s) can be found in the article/Supplementary material.

## Footnotes

1
http://www.rgenome.net/cas-designer/

## References

[B1] Ahmadi S. Yousif Abdullah B. Dlshad Taeeb S. Rashid Ahmed H. Majidi M. (2026). Alpha-pinene attenuates neuroinflammatory responses in the rat hippocampus and improves spatial working memory deficits associated with morphine dependence and withdrawal. *Behav. Brain Res.* 498:115920. 10.1016/j.bbr.2025.115920 41202988

[B2] Anderson R. M. Bitterman K. J. Wood J. G. Medvedik O. Cohen H. Lin S. S. et al. (2002). Manipulation of a nuclear NAD+ salvage pathway delays aging without altering steady-state NAD+ levels. *J. Biol. Chem.* 277 18881–18890. 10.1074/jbc.M111773200 11884393

[B3] Belcadi H. Chraka A. El Amrani S. Raissouni I. Moukhles A. Zantar S. et al. (2023). Investigation and valorization of the moroccan *Salvia Officinalis* L. essential oil: Phytochemistry, potential in corrosion inhibition, antibacterial activity, and theoretical modeling. *J. Bio Tribo Corros.* 9:50. 10.1007/s40735-023-00769-2

[B4] Ben Salha G. Abderrabba M. Labidi J. (2021). A status review of terpenes and their separation methods. *Rev. Chem. Eng.* 37 433–447. 10.1515/revce-2018-0066 31755547

[B5] Bhoir K. Prakash G. Odaneth A. (2025). Genetic engineering of *Yarrowia lipolytica* for 1,8-cineole production: A sustainable approach. *Enzyme Microb. Technol.* 189:110659. 10.1016/j.enzmictec.2025.110659 40273641

[B6] Blanchard L. Karst F. (1993). Characterization of a lysine-to-glutamic acid mutation in a conservative sequence of farnesyl diphosphate synthase from *Saccharomyces cerevisiae*. *Gene* 125 185–189. 10.1016/0378-1119(93)90326-x 8096487

[B7] Cao Y. Zhang H. Liu H. Liu W. Zhang R. Xian M. et al. (2018). Biosynthesis and production of sabinene: Current state and perspectives. *Appl. Microbiol. Biotechnol.* 102 1535–1544. 10.1007/s00253-017-8695-5 29264773

[B8] Cheah L. C. Liu L. Stark T. Plan M. R. Peng B. Lu Z. et al. (2023). Metabolic flux enhancement from the translational fusion of terpene synthases is linked to terpene synthase accumulation. *Metab. Eng.* 77 143–151. 10.1016/j.ymben.2023.03.012 36990382

[B9] Chen H. Su L. Yao Z. Jia K. Dai Z. Wang Q. (2026). Combinatorial engineering of enzyme and pathway for efficient β-farnesene bioproduction in *Yarrowia lipolytica*. *Synth. Syst. Biotechnol.* 12 32–41. 10.1016/j.synbio.2025.10.016 41321597 PMC12657309

[B10] Czajka J. J. Nathenson J. A. Benites V. T. Baidoo E. E. K. Cheng Q. Wang Y. et al. (2018). Engineering the oleaginous yeast *Yarrowia lipolytica* to produce the aroma compound β-ionone. *Microb. Cell Fact.* 17:136. 10.1186/s12934-018-0984-x 30172260 PMC6119263

[B11] Gao J. Li Y. Yu W. Zhou Y. J. (2022). Rescuing yeast from cell death enables overproduction of fatty acids from sole methanol. *Nat. Metab.* 4 932–943. 10.1038/s42255-022-00601-0 35817856

[B12] Gao L. Hou R. Cai P. Yao L. Wu X. Li Y. et al. (2024). Engineering yeast peroxisomes for α-bisabolene production from sole methanol with the aid of proteomic analysis. *JACS Au* 4 2474–2483. 10.1021/jacsau.4c00106 39055156 PMC11267555

[B13] Guo Y. Liao Y. Wang J. Ma C. Qin J. Feng J. et al. (2022). Methylotrophy of *Pichia pastoris*: Current advances, applications, and future perspectives for methanol-based biomanufacturing. *ACS Sustain. Chem. Eng.* 10 1741–1752. 10.1021/acssuschemeng.1c07755

[B14] Ignea C. Pontini M. Maffei M. E. Makris A. M. Kampranis S. C. (2014). Engineering monoterpene production in yeast using a synthetic dominant negative geranyl diphosphate synthase. *ACS Synth. Biol.* 3 298–306. 10.1021/sb400115e 24847684

[B15] Jiang G. Z. Yao M. D. Wang Y. Zhou L. Song T. Q. Liu H. et al. (2017). Manipulation of GES and ERG20 for geraniol overproduction in *Saccharomyces cerevisiae*. *Metab. Eng.* 41 57–66. 10.1016/j.ymben.2017.03.005 28359705

[B16] Jiang W. Newell W. Liu J. Coppens L. Borah Slater K. Peng H. et al. (2025). Insights into the methanol utilization capacity of Y. lipolytica and improvements through metabolic engineering. *Metab. Eng.* 91 30–43. 10.1016/j.ymben.2025.03.014 40158687

[B17] Juergens L. J. Worth H. Juergens U. R. (2020). New perspectives for mucolytic, anti-inflammatory and adjunctive therapy with 1,8-cineole in COPD and asthma: Review on the new therapeutic approach. *Adv. Ther.* 37 1737–1753. 10.1007/s12325-020-01279-0 32200535 PMC7467491

[B18] Krishanthi Abeywickrama A. A. C. K. A. Priyani P. Chammi S. Palehepitiya G. (2006). The efficacy of essential oil of *Alpinia calcarata* (Rosc.) and its major constituent, 1,8-cineole, as protectants of cowpea against *Callosobruchus maculatus* (F.) (Coleoptera: Bruchidae). *Can. J. Plant Sci.* 86 821–827. 10.4141/P04-013

[B19] Kubota K. Nakamura K. Murayama K. (1998). Acetoxy-1, 8-cineoles as aroma constituents of *Alpinia galanga* Willd. *J. Agric. Food Chem.* 46 5244–5247. 10.1021/JF9804239

[B20] Kuzman M. Sanvito L. Mitic B. M. Ata Ö. Mattanovich D. (2026). Rewiring methanol metabolism in *Komagataella phaffii* through implementation and evolution of a heterologous RuMP cycle. *Metab. Eng.* 96 28–38. 10.1016/j.ymben.2026.03.002 41791454

[B21] Li C. Yang Q. Huang Z. Yang Q. He W. Huang J. et al. (2026). Episomal plasmid engineering and pathway optimization for efficient 1,8-cineole production in *Yarrowia lipolytica*. *Green Chem.* 28 883–900. 10.1039/D5GC04873G

[B22] Lu Z. Peng B. Ebert B. E. Dumsday G. Vickers C. E. (2021). Auxin-mediated protein depletion for metabolic engineering in terpene-producing yeast. *Nat. Commun.* 12:1051. 10.1038/s41467-021-21313-1 33594068 PMC7886869

[B23] Martin V. J. Pitera D. J. Withers S. T. Newman J. D. Keasling J. D. (2003). Engineering a mevalonate pathway in *Escherichia coli* for production of terpenoids. *Nat. Biotechnol.* 21 796–802. 10.1038/nbt833 12778056

[B24] Nan C. Guo X. Cheng J. Li X. Feng J. Wang J. et al. (2026). *De novo* biosynthesis of sabinene from methanol by multiple engineered *Pichia pastoris*. *J. Agric. Food Chem.* 74 5405–5416. 10.1021/acs.jafc.5c12464 41645819

[B25] Peng B. Nielsen L. K. Kampranis S. C. Vickers C. E. (2018). Engineered protein degradation of farnesyl pyrophosphate synthase is an effective regulatory mechanism to increase monoterpene production in *Saccharomyces cerevisiae*. *Metab. Eng.* 47 83–93. 10.1016/j.ymben.2018.02.005 29471044

[B26] Peng B. Plan M. R. Chrysanthopoulos P. Hodson M. P. Nielsen L. K. Vickers C. E. (2017). A squalene synthase protein degradation method for improved sesquiterpene production in *Saccharomyces cerevisiae*. *Metab. Eng.* 39 209–219. 10.1016/j.ymben.2016.12.003 27939849

[B27] Qi M. Zhu C. Cheng C. Kang W. Xue C. (2025). Rewiring methanol assimilation and reductive glycine pathways in *Saccharomyces cerevisiae* to increase one-carbon recovery. *Green Chem.* 27 3261–3271. 10.1039/D4GC05254D

[B28] Ro D. K. Paradise E. M. Ouellet M. Fisher K. J. Newman K. L. Ndungu J. M. et al. (2006). Production of the antimalarial drug precursor artemisinic acid in engineered yeast. *Nature* 440 940–943. 10.1038/nature04640 16612385

[B29] Rusz M. Del Favero G. El Abiead Y. Gerner C. Keppler B. K. Jakupec M. A. et al. (2021). Morpho-metabotyping the oxidative stress response. *Sci. Rep.* 11:15471. 10.1038/s41598-021-94585-8 34326354 PMC8322264

[B30] Rußmayer H. Buchetics M. Gruber C. Valli M. Grillitsch K. Modarres G. et al. (2015). Systems-level organization of yeast methylotrophic lifestyle. *BMC Biol.* 13:80. 10.1186/s12915-015-0186-5 26400155 PMC4580311

[B31] Sakamaki Y. Ono M. Shigenari N. Chibazakura T. Shimomura K. Watanabe S. (2023). Photosynthetic 1,8-cineole production using cyanobacteria. *Biosci. Biotechnol. Biochem.* 87 563–568. 10.1093/bbb/zbad012 36810583

[B32] Schrader J. Schilling M. Holtmann D. Sell D. Filho M. V. Marx A. et al. (2009). Methanol-based industrial biotechnology: Current status and future perspectives of methylotrophic bacteria. *Trends Biotechnol.* 27 107–115. 10.1016/j.tibtech.2008.10.009 19111927

[B33] Shaw J. J. Berbasova T. Sasaki T. Jefferson-George K. Spakowicz D. J. Dunican B. F. et al. (2015). Identification of a fungal 1,8-cineole synthase from *Hypoxylon* sp. with specificity determinants in common with the plant synthases. *J. Biol. Chem.* 290 8511–8526. 10.1074/jbc.M114.636159 25648891 PMC4375501

[B34] Sonntag F. Kroner C. Lubuta P. Peyraud R. Horst A. Buchhaupt M. et al. (2015a). Engineering *Methylobacterium extorquens* for *de novo* synthesis of the sesquiterpenoid α-humulene from methanol. *Metab. Eng.* 32 82–94. 10.1016/j.ymben.2015.09.004 26369439

[B35] Sonntag F. Müller J. E. Kiefer P. Vorholt J. A. Schrader J. Buchhaupt M. (2015b). High-level production of ethylmalonyl-CoA pathway-derived dicarboxylic acids by *Methylobacterium extorquens* under cobalt-deficient conditions and by polyhydroxybutyrate negative strains. *Appl. Microbiol. Biotechnol.* 99 3407–3419. 10.1007/s00253-015-6418-3 25661812

[B36] Vickers C. E. Williams T. C. Peng B. Cherry J. (2017). Recent advances in synthetic biology for engineering isoprenoid production in yeast. *Curr. Opin. Chem. Biol.* 40 47–56. 10.1016/j.cbpa.2017.05.017 28623722

[B37] Wang C. Gou L. Liu D. Wu S. Zhou X. Fan T.-P. et al. (2026). Rational enzyme modification and expression enhancement strategies for 1,8-cineole production in *Serratia marcescens* cell factories. *Appl. Biochem. Biotech.* 198 1619–1634. 10.1007/s12010-025-05544-2 41484735

[B38] Wang C. Yoon S. H. Jang H. J. Chung Y. R. Kim J. Y. Choi E. S. et al. (2011). Metabolic engineering of *Escherichia coli* for α-farnesene production. *Metab. Eng.* 13 648–655. 10.1016/j.ymben.2011.08.001 21907299

[B39] Wang H. Bai F. Lu H. Zhou Y. J. (2025). Understanding methanol metabolism through systems biology: Advances and future perspectives. *Curr. Opin. Biotechnol.* 96:103370. 10.1016/j.copbio.2025.103370 41086471

[B40] Wit N. Gogola E. West J. A. Vornbäumen T. Seear R. V. Bailey P. S. J. et al. (2023). A histone deacetylase 3 and mitochondrial complex I axis regulates toxic formaldehyde production. *Sci. Adv.* 9:eadg2235. 10.1126/sciadv.adg2235 37196082 PMC10191432

[B41] Xing P. Duan J. Q. Gao Y. J. Zhou J. (2018). Advances in engineering methylotrophic yeast for biosynthesis of valuable chemicals from methanol. *Chin. Chem. Lett.* 29 45–50. 10.1016/j.cclet.2017.11.015

[B42] Yasokawa D. Murata S. Iwahashi Y. Kitagawa E. Nakagawa R. Hashido T. et al. (2010). Toxicity of methanol and formaldehyde towards *Saccharomyces cerevisiae* as assessed by DNA microarray analysis. *Appl. Biochem. Biotechnol.* 160 1685–1698. 10.1007/s12010-009-8684-y 19499198

[B43] Ye C. Li M. Gao J. Zuo Y. Xiao F. Jiang X. et al. (2024). Metabolic engineering of *Pichia pastoris* for overproduction of cis-trans nepetalactol. *Metab. Eng.* 84 83–94. 10.1016/j.ymben.2024.06.007 38897449

[B44] Zang X. Yang Y. Zhan C. Bai Z. (2025a). Blocking methanol dissimilation pathway and incorporating RuMP to increase methanol utility in *Komagataella phaffii*. *Syst. Microbiol. Biomanuf.* 5 1272–1285. 10.1007/s43393-025-00356-1

[B45] Zang X. Yang Y. Zhan C. Bai Z. (2025b). Methanol metabolism in synthetic methylotrophic microorganisms. *Biotechnol. Adv.* 83:108623. 10.1016/j.biotechadv.2025.108623 40499848

[B46] Zhan C. Li X. Yang Y. Nielsen J. Bai Z. Chen Y. (2021). Strategies and challenges with the microbial conversion of methanol to high-value chemicals. *Biotechnol. Bioeng.* 118 3655–3668. 10.1002/bit.27862 34133022

