# Supplementary material for: Metabolic engineering of Pichia pastoris for sustainable production of 1,8-cineole from methanol
Source: Front Microbiol. 2026 Jun 19;17:1866106. doi: 10.3389/fmicb.2026.1866106 (PMC13328473; doi:10.3389/fmicb.2026.1866106)
Supplement: Supplementary file 1 [file Data_Sheet_1.pdf]

Supporting Information

Metabolic Engineering of *Pichia pastoris* for Sustainable Production of 1,8-cineole from Methanol

Cui Zhao[1], Tengfei Wang[2, 3], Hongling Liu[2, 3],Yanpo Li[1], Weiwei Wang[1], XiHui Wang[1]*
[1] Food Nutrition and Health Research Laboratory, School of Health and Elderly Care, Shandong Women's University, Jinan 250300, China
[2] State Key Laboratory of Biobased Material and Green Papermaking (LBMP), Qilu University of Technology (Shandong Academy of Sciences), Jinan 250353, China
[3] Key Laboratory of Shandong Microbial Engineering, College of Bioengineering, QiLu University of Technology (Shandong Academy of Sciences), Jinan 250353, China
*Corresponding author: XiHui Wang (wxh1991yswh@163.com)

Table S1 The main plasmids used in this study

Plasmids	Inserted fragment
pICZ-*CnSA*	*PNSI-1::*P_{AOX1}-*CnSA*-T_{AOX1}
pICZ-*Hyp3*	*PNSI-1::*P_{AOX1}-*Hyp3*-T_{AOX1}
pICZ-*Erg20*^{ww}	*PNSII-1::*P_{DAS2}-*Erg20*^{ww}-T_{AOX1}
pICZ-*Erg20*^{ww}-*Hyp3*	P_{DAS2}-*Erg20*^{ww}-*Hyp3*-T_{AOX1}
pICZ-*Hyp3*-*Erg20*^{ww}	*PNSII-1::*P_{DAS2}-*Hyp3*-*Erg20*^{ww}-T_{AOX1}
pICZ-*Erg10*-*Erg13*	*PNSII-8::*P_{TDH3}-*Erg10*-T_{AOX1}-P_{TEF1}-*Erg13*-T_{AOX1}
pICZ-*tHMG1*	*PNSIV-7::*P_{GAP1}-*tHMG1*-T_{AOX1}
pICZ-*Erg12*-*Erg8*-*Erg19*	*PNSIII-6::*P_{ADH2}-*Erg12*-T_{GAP1}-P_{TEF1}-*Erg8*-T_{DAS1}-P_{GAP1}-*Erg19*-T_{AOX1}
pICZ-*IDI1*	*PNSII-5::*P_{GPM1}-*IDI1*-T_{AOX1}
pICZ-*AOX1*-*DAS2*	*PNSIII-8::*P_{ADH2}-*AOX1*-T_{GAP1}-P_{TEF1}-*DAS2*-T_{TEF1}
pICZ-*ZWF1-SOL2-GND1*	*PNSIV-12::*P_{TEF1}-*ZWF1*-T_{TEF1}-P_{AOX1}-*SOL2*-T_{DAS1}-P_{DAS2}-*GND1*-T_{AOX1}
pICZ-*RPE1-TAL1-TKL1*	*PNSIV-12::*P_{TEF1}-*RPE1*-T_{TEF1}-P_{AOX1}-*TAL1*-T_{DAS1}-P_{DAS2}-*TKL1*-T_{AOX1}
pICZ-*ADE3-SHM2*	*FDH::*P_{AOX1}-*ADE3*-T_{TEF1}-P_{AOX1}-*SHM2*-T_{DAS1}

Table S2 The main primers used in this study

Name	Primer sequence （5'-3'）
GAP_F	CACCACAGCAGCACCAACAG
AOX1_R	TCTCACTTAATCTTCTGTAC
CnSA_F	CATAGAGAAGAGAATCTTTATCTATCTCACAGAAAAGAGAAT CTACGACCATGCCCGCCGGCCACGAAGA
CnSA_R	GTTTTTTTTTTCTTTTTCGATAGTGTATATTAAGGTGTAGAAGA ATAGTGTCACCAAGGGGTGGTGGCCC
*Hyp3*_F	TGGGCAACTGTGTTTAGCCATATAATAAGAGACACTATTACAC TTACACAATGCGGCCCATCACTTGTTC
*Hyp3*_R	CGTATTTGTGCTGTCCTGCTCACGAAAAGGCACCAAGGACAT GTCTCTATCTAGATACCACGCAGCCCCG
Erg20^{ww}_F	AACAAACACAATTACAAAAAATGAAGAACTACTTCCCAAAC
Erg20^{ww}_R	GCATTCTGACATCCTCTTGAATCTGAACAAGATGTTGTTG
ERG20^{ww}_*Hyp3*_F	GCATTCTGACATCCTCTTGAATATGCGGCCCATCACTTGTTC
ERG20^{ww}_*Hyp3*_R	CTAGATACCACGCAGCCCCGTAAAAACATGGGGTAAAAA
*Hyp3*_ERG20^{ww}_F	TGAAGAACTACTTCCCAAACATGCGGCCCATCACTTGTTC
*Hyp3*_ERG20^{ww} _R	CTGAACAAGATGTTGTTGCTAGATACCACGCAGCCCCG
tHMGR_F	AACAAACACAATTACAAAAAATGTCAAAGAAGACCAAGAA
tHMGR_R	GCATTCTGACATCCTCTTGATTACGACCGAATACAAATCT
IDI1_F	AACAAACACAATTACAAAAAATGAGTTTGTCAGCCTACCA
IDI1_R	GCATTCTGACATCCTCTTGATTAGAGCATACGATCAATAT
*Erg10*_F	AACAAACACAATTACAAAAAATGAGTGAACCTGTTTACAT
*Erg10*_R	GCATTCTGACATCCTCTTGATTCAAATATGACTCTTTGAGG
*Erg13*_F	AACAAACACAATTACAAAAAATGTCTCGTCCAAGTAACAT
*Erg13*_R	GCATTCTGACATCCTCTTGATTTAGTTTTTAACCTGGTATT
*Erg19*_F	AACAAACACAATTACAAAAAATGTGTCTTCAAAGTATCGT
*Erg19*_R	GCATTCTGACATCCTCTTGATTTAAGGAGCAACTAAAAAC
*Erg12*_F	AACAAACACAATTACAAAAAATGAAAGCTTTCAGCGCTCC
*Erg12*_R	GCATTCTGACATCCTCTTGATCTATTCTGCAAATAAATAGC
*Erg8*_F	AACAAACACAATTACAAAAA ATGTGGCTACCTTGTTCTTG
*Erg8*_R	GCATTCTGACATCCTCTTGATCTAGATAATATCGTCGATGG
*DAS2*_F	AACAAACACAATTACAAAAAATGGACAGAAAAGCTGTCGA
*DAS2*_R	GCATTCTGACATCCTCTTGATGATATTACGATCTGTCATGA
*AOX1*_F	AACAAACACAATTACAAAAAATGGCTATCCCCGAAGAGTT
*AOX1*_R	GCATTCTGACATCCTCTTGATTTAGAATCTAGCAAGACCGG
*ZWF1*_F	AACAAACACAATTACAAAAAATGGCTATCCCCGAAGAGTT
*ZWF1*_R	GCATTCTGACATCCTCTTGATTTAGAATCTAGCAAGACCGG
*GND1*_F	AACAAACACAATTACAAAAAATGGTTGAAGCAACAGGAGA
*GND1*_R	GCATTCTGACATCCTCTTGATTTAAGCATCGTAGGTACTGG

*SOL2*_F	AACAAACACAATTACAAAAAATGGTACAAATCTATTCCTA
*SOL2*_R	GCATTCTGACATCCTCTTGATTCAGTATTTCGAAGTAGAAA
*RPE1*_F	AACAAACACAATTACAAAAAATGGTCAAACCAATTATAGC
*RPE1*_R	GCATTCTGACATCCTCTTGATCTAATCTAGCAAATCTCTAG
*TAL1*_F	AACAAACACAATTACAAAAAATGGAATCCAATCCTATCAA
*TAL1*_R	GCATTCTGACATCCTCTTGATTTACAACTTGGATAAAACAC
*TKL1*_F	AACAAACACAATTACAAAAAATGGCTAGAATTCCCAAAGC
*TKL1*_R	GCATTCTGACATCCTCTTGATTTATAGTTTGTCGTGCTTTG
*ADE3*_F	AACAAACACAATTACAAAAAATGGCTGGTCAAGTGTTGGAC GGCAAAGCATGCGC
*ADE3*_R	AACAAACACAATTACAAAAATTAGAACAGGCCATCGATCTC ACCGTCATCATCGA
*SHM2*_F	AACAAACACAATTACAAAAAATATGTTGTTTACCAACGTAAC GCGTGTTTTCGCTTC
*SHM2*_R	GCATTCTGACATCCTCTTGATGGTATGTAAACTGAAATGCGTT TAATGGCTTAACT

Table S3 Sequences of synthetic genes used in this study

Gene name	Nucleotide sequences (5’ - 3’)
CnSA<br>(GenBank:<br>CP045850.1)	ATGCCCGCCGGCCACGAAGAGTTCGACATACCCTTCCCGTCCCGAGT<br>GAACCCCTTCCACGCCCGGGCCGAGGACCGCCATGTGGCCTGGATGC<br>GCGCCATGGGTCTGATCACCGGCGACGCCGCCGAAGCGACGTACCGC<br>CGCTGGAGTCCCGCCAAGGTCGGAGCCCGCTGGTTCTATCTCGCACA<br>GGGTGAGGACCTGGACCTGGGCTGCGACATCTTCGGCTGGTTCTTCGC<br>CTATGACGACCACTTCGACGGACCCACCGGCACCGATCCCCGGCAGA<br>CCGCCGCGTTCGTGAACCGCACCGTGGCCATGCTCGACCCCCGCGCC<br>GACCCGACCGGCGAGCACCCCCTCAACATCGCCTTTCACGACTTATGG<br>CAACGTGAGAGCGCCCCCATGTCCCCCCTCTGGCAGCGGCGTGCCGT<br>CGACCACTGGACGCAGTACCTCACCGCCCACATCACCGAGGCGACCA<br>ACCGCACCCGCCACACCAGTCCCACCATCGCCGACTACCTGGAGCTG<br>CGCCACCGCACCGGCTTCATGCCCCCGCTGCTCGACCTGATCGAACGG<br>GTCTGGCGCGCCGAAATCCCCGCCCCCGTCTACACCACCCCCGAAGT<br>GCAGACCCTTCTCCATACGACCAACCAGAACATCAACATCGTCAATG<br>ACGTCCTGTCCCTGGAGAAGGAAGAAGCCCACGGCGACCCGCACAAC<br>CTCGTCCTGGTCATCCAGCACGAGCGGCAGAGTACCCGACAGCAGGC<br>CCTCGCCACCGCCCGGCGCATGATCGACGAGTGGACCGATACCTTCA<br>TCCGCACCGAACCCCGGCTCCCCGCCCTCTGCGGCCGCCTCGGCATTC<br>CTCTCGCCGACCGCACATCCCTCTACACCGCCGTCGAAGGCATGCGCG<br>CCGCCATCCGCGGCAACTACGACTGGTGCGCCGAGACCAACCGCTAC<br>GCCGTTCACCGCCCCACCGGCACGGGGCGGGCCACCACCCCTTGGTG<br>A
Hyp3<br>(GenBank:<br>KJ433271.1)	ATGCGGCCCATCACTTGTTCATTTGACCCAGTTGGGATCTCCTTTCAGA<br>CGGAGTCGAAGCAAGAGAACTTCGAATTCTTAAGAGAGGCTATCTCTC<br>GCTCTGTACCTGGCCTAGAAAATTGCAATGTCTTTGACCCTCGCTCCTT<br>AGGAGTACCATGGCCGACATCATTTCCCGCCGCAGCACAGAGCAAGTA<br>CTGGAAGGATGCGGAAGAAGCGGCAGCAGAATTAATGGACCAAATCG

	TCGCCGCAGCGCCGGGCGAGCAAGGCTCATTACCAGCAGAGTTGGCA<br>GTCTCAGATAAGAAAGCAGCCAAGAGACGAGAGCTACTCGACACATC<br>TGTTTCGGCGCCGATGAACATGTTTCCTGCGGCAAACGCCCCGCGGGC<br>GAGAATAATGGCAAAGGCAAATTTGCTAATCTTCATGCATGACGGTAA<br>GCTGCTTTCGTGTTTCACCAGAAACTGCAAACCACATACCTTATAGCAT<br>TCTATACGAATGTTTATACCTCGGACTCTAGAGGCTCTTCCTCTTTAATC<br>TATTAAAAGTGATTACACGGGACGCTAACAACAGTATACAGACGTCTG<br>CGAGTATCAGTCCGTGGTATGTGGTATTATTGAAGTCCTGCGATGAGAT<br>TGTTAACACTTGATACTAGCAATCGACAATTATCGACTCTGCGCTTGCT<br>GATACCAGCACTCCTAATGGGAAAGGCGCAGACATACTATGGCAAAAC<br>AGGATCTTCAAAGAGTTTTCAGAGGAAACCAACAGAGAAGATCCCGT<br>TGTGGGACCCCAATTCCTCCAAGGTATATTGAATTGGGTAGAACACACC<br>CGCAAGGCGCTGCCCGCTTCCATGACTTTCCGCTCTTTCAACGAGTAC<br>ATCGACTACCGCATCGGGGACTTCGCTGTGGAGTAAGTCTTAAGTCTC<br>AGTTTGACATAGTAATGCGGCGCAGCTAACGACGAATAGCTTTTGCGAT<br>GCAGCTATCTTGCTGACTTGTGAGATTTTCCTGACTCCAGCCGATATGG<br>AGCCCCTCAGGAAATTACATCGACTTTACATGACTCACTTCTCGTTAAC<br>CAACGATTTATACTCCTTTAATAAGGAGGTTGTAGCGGAGCAGGAAAC<br>CGGCTCCGCAGTGATCAATGCTGTCAGAGTCCTAGAACAGCTTGTCGA<br>TACTTCAACTCGATCAGCCAAAGTCCTTCTTAGAGCATTCCTGTGGGAT<br>CTCGAACTACAAATACACGACGAGCTCACGCGGCTCAAAGGTACAGA<br>CCTCACTCCCAGTCAATGGCGCTTCGCCCGCGGTATGGTAGAGGTGTG<br>TGCGGGAAATATATTCTACTCCGCAACTTGCTTGCGGTATGCTAAACCG<br>GGGCTGCGTGGTATCTAG

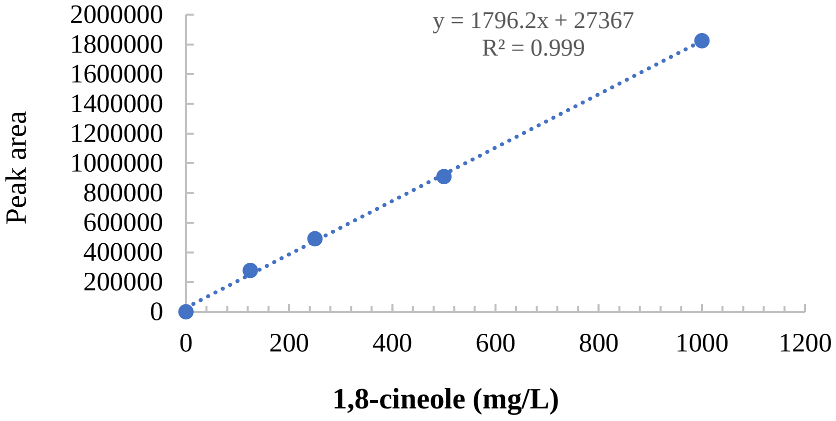

Figure S1. Standard curve for 1,8-cineole quantification. All values represent the means of 3 replicates.

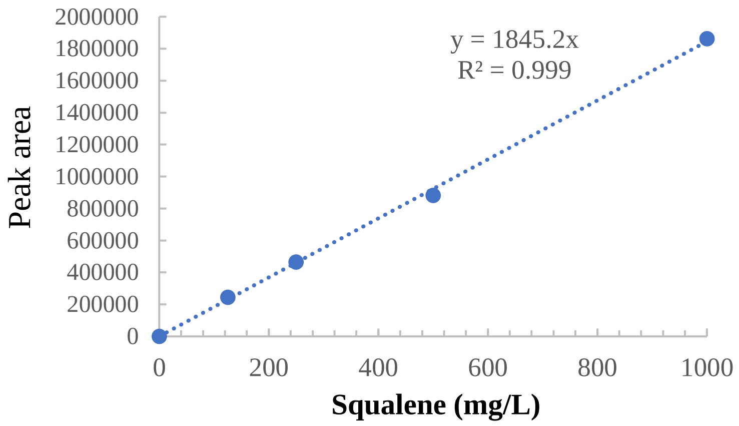

Figure S2. Standard curve for squalene quantification. All values represent the means of 3 replicates.

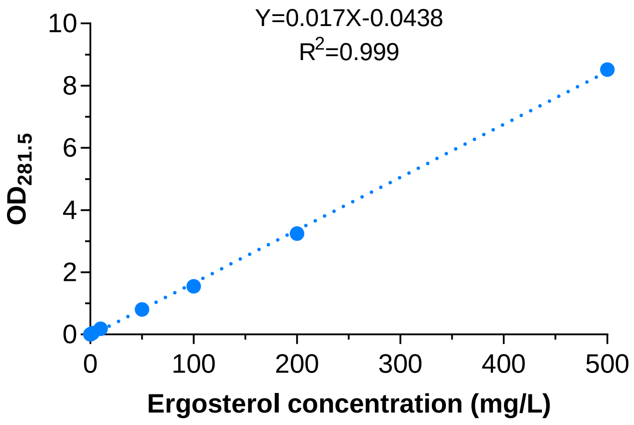

Figure S3. Standard curve for ergosterol quantification. All values represent the means of 3 replicates.

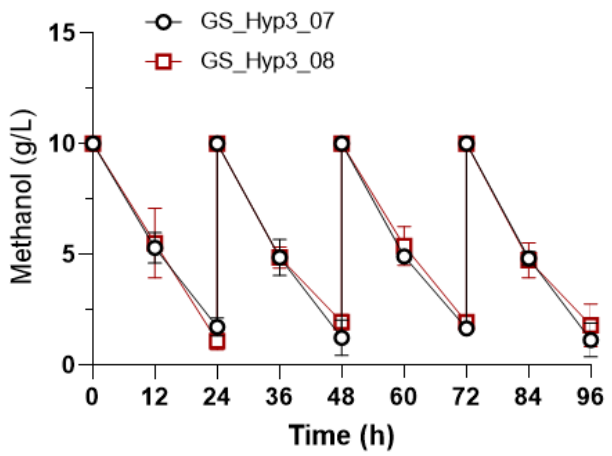

Figure S4. The methanol consumption of the engineered strains GS_Hyp3_07 and GS_Hyp3_08. All values represent the means of 3 replicates.

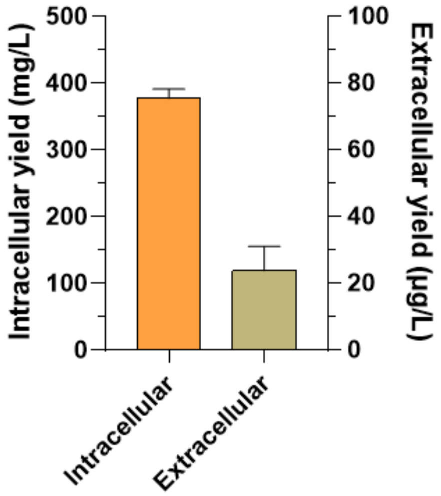

Figure S5. The intracellular and extracellular levels of 1,8-cineole in the engineered strain GS_Hyp3_12 in a 5-L bioreactor. These data represent average values and standard deviations achieved from three independent experiments.
